# Decision Making and Oddball Effects on Pupil Size: Evidence for a Sequential Process

**DOI:** 10.5334/joc.96

**Published:** 2020-03-27

**Authors:** Christoph Strauch, Ina Koniakowsky, Anke Huckauf

**Affiliations:** 1 General Psychology, Ulm University, DE

**Keywords:** Pupil dilation, reaction times, decision making, oddball, stimulus relevance

## Abstract

In our physical environment as well as in many experimental paradigms, we need to decide whether an occurring stimulus is relevant to us or not; further, stimuli have uneven probabilities to emerge. Both, decision making and the difference between rare and frequent stimuli (oddball effect) are described to affect pupil dilation. Surprisingly though, conjoint systematic pupillometric investigations into both factors are still rare. In two experiments, both factors as well as their interplay were investigated. Participants completed a sequential letter matching task. In this task, stimulus probability and letter matching (decision making) were manipulated independently. As dependent variables, pupil dilation and reaction time were assessed. Results suggest a clearly larger pupil dilation for target than for distractor letters, even when targets were frequent and distractors rare. When considering the data structure best, no main effect of stimulus probability was found, instead, oddball effects only emerged when stimuli were goal-relevant to participants. The results are discussed in the light of common theoretical concepts of decision making and stimulus probability. Finally, relating theories of each factor, we propose an integrated framework for effects of decision making and stimulus features on pupil dilation. We assume a sequential mechanism during which incoming stimuli are decided upon regarding their goal relevance and, about 200 ms later, relevant stimuli are appraised regarding their value.

## Introduction

All behavior may be conceived as being based upon decisions, be it applying for a job, a studies program, or a simple choice, such as to whether drinking black or green tea. Whatever decision is reached at a given point in time, it will shape upcoming cognition and behavior. Understanding cognition and behavior may be considered one, if not the principal aim of psychological research. Hence, discerning ongoing decision processes or, even further, detecting and predicting chosen options is fundamental for understanding what cognition and behavior are performed. The way decisions are formed is thus an area of ongoing intense research interest. Besides analyzing behavioral measures, such as reaction times, psychophysiological indicators have become increasingly popular, as they may provide more fine grained information on how decision processes unfold (Satterthwaite et al., 2007, e.g.). As such, pupil dilation has been demonstrated to allow for investigating decision making (De Gee, Knapen, & Donner, 2014).

Moreover, pupils dilate differentially when processing rare stimuli in comparison to frequent ones (so called oddball effects). Pursuing a specific goal may often include a specific desired stimulus that has to be selected from a larger set of irrelevant stimuli, e.g., a specific key needs to be selected from your key ring to open a specific door. Or, similarly, when waiting at the bus stop for a specific line, possibly several buses will go by before the bus to get on arrives; arriving buses have a certain probability to be the bus to get on. When going through the options, be it by gaze, by memory, or else, each incoming stimulus will be decided upon until the desired option is found.

In the above example, as in most other situations, effects of decision making and processing probability coincide, disentangling them is thus challenging, but relevant for theoretical considerations. For observed effects, the question arises what part of a physiological or behavioral response may be attributed to decision making and what part to stimulus probability. These questions might be answered using pupil dilation. Results on the interplay of decision making and stimulus probability however, are yet sparse and the only investigation using pupil dilation (Qiyuan, Richer, Wagoner, & Beatty, 1985) is contradictory to recent findings from decision making under equiprobability (De Gee et al., 2014; Strauch, Greiter, & Huckauf, 2018). Investigating commonalities and differences of effects ascribed to decision making and to stimulus probability, two experiments were conducted using a sequential letter selection paradigm that allows for manipulating both factors independently.

In the following sections, we introduce pupillometry as psychophysiological measure. Subsequently, literature on decision making and on processing stimulus probability is introduced by example, resulting in a description of the interplay of decision making and stimulus probability.

### Pupillometry

Besides changes in brightness or due to accomodation, pupil diameter reflects fluctuations in central nervous activation. While the precise neural connections are still not fully understood, variations in pupil size are linked to activity in Locus Coeruleus (LC) and the associated norepinephrine (NE) system in a high temporal resolution (Joshi, Li, Kalwani, & Gold, 2016). A variety of psychological processes has been investigated more closely using pupil diameter, ranging from cognitive load (Hess & Polt, 1964, e.g.) over emotional activation (Ehlers, Strauch, Georgi, & Huckauf, 2016, e.g.), reward-anticipation (Schneider, Leuchs, Czisch, Sämann, & Spoormaker, 2018), relevance (Simpson, 1969) and self-relevance (Greiter, Strauch, & Huckauf, 2018), decision making (De Gee et al., 2014, e.g.), to effects of stimulus probability (Murphy, O’Connell, O’Sullivan, Robertson, & Balsters, 2014) and beyond (see Einhäuser (2017) and Mathôt (2018) for reviews).

Pupil diameter is not the only psychophysiological indicator affected by changes in arousal or the LC-NE system. Especially effects in the P3 ERP component have been described to align with changes in pupil size elicited by fluctuations in arousal accompanying cognition (Nieuwenhuis, De Geus, & Aston-Jones, 2011). At the time of the review of Nieuwenhuis et al. (2011), effects of stimulus probability had been described for EEG and for pupil diameter. Effects of deciding between equiprobable stimuli that have been regularly described for the P3, however, were very limited for pupil diameter. Meanwhile, results show that target stimuli indeed elicit a stronger pupil dilation than distractors in equiprobable paradigms (De Gee et al., 2014; Strauch et al., 2018, e.g.). Still, as of today, the interplay of stimulus probability and decision making in their effects on pupil size has not been further investigated. In the following two paragraphs, we shortly introduce existing literature on decision making/choice and oddball effects, as well as their interaction for pupil dilation, here we will also describe effects for the P3, as pupillometric investigations are yet scarce.

### Decision making: the choice effect

In most existing research, decision making is investigated with a focus on binary choice and therefore rather represents *choosing* between two options or ‘yes’ and ‘no’ respectively. As one of the first groups, Simpson and Hale (1969) demonstrated that pupils dilate more when participants decide to move a lever in a 2-choice decision task compared to participants instructed to move a lever without a decision task. This effect was ascribed to the cognitive load elicited by the decision process. Similarly, van Olst, Heemstra, and Ten Kortenaar (1979) and Simpson (1969) report a larger pupil dilation for tones that required a key press compared to those with no key press; they ascribe these effects to the decision between options differing in stimulus relevance. Further, it has been demonstrated that not only decision making per se, but also the outcome of the decision making process affects pupil size: three different options in a letter matching task could be distinguished using pupil diameter (Beatty & Wagoner, 1978). More recently, Einhäuser, Koch, and Carter (2010) presented a sequence of numbers to participants with the instruction to push a lever, when participants wanted to select a number. The selected numbers were associated with a larger pupil diameter than rejected numbers. Further, Einhäuser et al. (2010) required participants to indicate the selected number after the presentation of all numbers in a second investigation. Still, the selected number was associated with a larger pupil diameter than other numbers; however, with a comparably small effect size. Here, targets were always rare. Similarly, in visual search tasks, fixations on targets are accompanied by a larger pupil dilation than fixations on more frequent distractors (Klingner 2010). However, when targets and distractors are unbalanced, observed effects may be due to either decision making or processing differing stimulus probabilities.

In recent years, pupil responses were also demonstrated repeatedly to be stronger for targets than for distractors in equiprobable setups: Employing a signal detection task, where each trial had to be answered via key press, a consistent choice effect could be demonstrated (De Gee et al., 2017, 2014), i.e., pupil dilated more for ‘yes’ than ‘no’ responses. Employing letter selection, Strauch et al. (2018) demonstrated a stable larger pupil dilation for target compared to distractor letters.

### Stimulus probability: the oddball effect

In a stream of distractor stimuli, low probability target stimuli (*oddballs*) elicit a differential physiological (e.g. pupil dilation) and behavioral response (e.g. reaction times). A stronger pupil dilation is found, the rarer the target stimulus: Rare target stimuli requiring a response in a visual oddball task elicited a larger pupillary response than frequent stimuli (Murphy, O’Connell, et al., 2014). Similar findings have been described also for the auditory modality (Gilzenrat, Nieuwenhuis, Jepma, & Cohen, 2010). In line with this finding, rarely used words are associated with larger pupil responses than frequently used words, showing the oddball effect also for more complex stimulus material (Kuchinke, Võ, Hofmann, & Jacobs, 2007). Hereby, pupil dilation is inversely related to stimulus probability (Gilzenrat et al., 2010; Qiyuan et al., 1985, e.g.). Still, such effects may also result from motor execution affecting pupil dilation (Richer & Beatty, 1985), coinciding with target but not distractor stimuli. To our knowledge, no oddball investigation using pupillometry exists that controls for the effects of motor execution.

### Interplay of decision making and stimulus probability

Understanding better how we choose, but also how pupils dilate when processing different options, requires understanding how deciding interacts with stimulus probability: In our everyday lives, choosing between equally probable options represents rather exception than norm. Main effects ascribed to decision making or the oddball effect may often carry components of both: Many paradigms investigating the effects of stimulus probability entangle these effects with decision making by assigning relevance to (most often rare) stimuli. When effects of stimulus probability become visible, stimuli are typically connected to a meaningful consequence, for example, a monetary reward or punishments for errors (Verleger, Cäsar, & Smigasiewicz, 2017, e.g.). These stimuli then have to be counted or responded to via key press or by moving a lever.

For EEG, a number of investigations pursued the interaction of decision making and stimulus probability. Besides demonstrating the oddball effect, Duncan-Johnson and Donchin (1977) show P3 to be more accentuated for target compared to distractor stimuli even when equiprobable, which hints at the relevance of a stimulus for a task as underlying mechanism. Bruin and Wijers (2002) report that also when target stimuli had to be counted instead of indicated via key response, they elicited a stronger P3 than distractor stimuli. The P3 was stronger, even when targets were more probable than distractor stimuli. While concluding that the oddball effect may not simply be traced back to movement (as effects were also visible in the count condition), this suggests that stimulus category (target/distractor) elicits a stronger response than stimulus probability and both effects come into place simultaneously.

For pupil dilation however, only one investigation explicitly examining the interplay of choosing and stimulus probability is known to the authors: Qiyuan et al. (1985) report pupil dilation to be the stronger, the rarer stimuli were in a stream of differentially pitched sounds. However, contradicting investigations into decision making solely under equiprobability (De Gee et al., 2014; Simpson, 1969; Strauch et al., 2018, e.g), targets (that had to be counted) and distractors were associated to almost identical pupil sizes. Thus, differing from the P3 in Bruin and Wijers (2002, e.g.), targets elicited a smaller pupil dilation than distractors when targets were frequent (Qiyuan et al., 1985).

Therefore, we reinvestigated the interplay of decision making (choice) and stimulus probability on pupil dilation in two experiments. Hereby, both the absolute size of a pupil change and the temporal succession of pupil diameter changes are of interest. In addition to changes in psychophysiology, many of the aforementioned effects may also become visible in reaction times. Generally, reaction times are described to be shorter to targets than to distractors (De Gee et al., 2014, e.g.) and shorter for frequent compared to rare stimuli (Gordon, 1967, e.g.). In existing oddball literature, reaction times are usually assessed for targets only (Murphy, O’Connell, et al., 2014, e.g.), which allows investigating the effects of stimulus probability, but neglects potential effects of decision making and their interaction. Therefore, in both experiments, we assessed reaction times for rare, equiprobable and frequent stimuli as well as their combinations with target or distractor independent from either of those categories. The combination of pupil dilation and reaction times further allows for discussing the results in light of common theories on decision making and stimulus probability and pupil dilation in general (here: drift diffusion model, response facilitation, stimulus-response pairing, goal-relevance).

## Experiment 1

In Experiment 1, the primary research aim/question was to replicate effects of choice and of stimulus probability with a paradigm allowing for assessing pupil dilation and reaction time. In this experiment we employed a letter matching paradigm as described in Strauch et al. (2018), also, the interaction effect was investigated.

### Methods

Data for all reported effects may be retrieved in conjunction with short videos of a series of trials and further supplementary documents via the open science framework (https://osf.io/yfszq/).

#### Participants

Fifty-six participants took part in Experiment 1 (*M*_Age_ = 22.19 years, 45 female). Due to technical errors, data were not recorded for 12 participants in Experiment 1 for at least one block, which is why these participants’ data did no contribute to the analyses. Data generated by the remaining 44 participants were subsequently used for statistical analyses for the respective experiments. All participants had normal or corrected to normal vision.

#### Apparatus

Participants were seated at 60 cm distance from eye position to a 27-inch screen with a resolution of 1920*1080 px and a refresh rate of 144 Hz, on which the experiments were presented. Luminance at eye position was kept constant at 60 lx. For all experiments, a SMI Hi-Speed 1250 Eye tracker (SensoMotoricInstruments GmbH) was used, including a chin rest.

#### Design

In Experiment 1, the factors stimulus probability (25%, 50%, 75%), choice (target/distractor) and GoNoGo (Go/NoGo) were investigated, resulting in a 3 × 2 × 2 repeated measures design. Hereby, GoNoGo was varied randomly, i.e. at a chance of 50%, a tone prompted participants to indicate the correctness of a letter via key press (Go), else, no prompt was played and no key press required (NoGo). Stimulus probability was manipulated in a blockwise manner and either set to 25%, 50% or 75% chance for a target stimulus to be presented in each trial compared to a distractor stimulus. The order of the three blocks was kept balanced between participants using a latin square (25%–50%–75%: n = 15; 50%–25%–75%: n = 15; 75%–50%–25%: n = 14). For both experiments, pupil dilation and reaction times (Go trials only) were assessed as dependent variables.

#### Procedure and task

After giving written informed consent and filling in a demographic data sheet (age, gender), participants were instructed about the task by the experimenter as well as reading an instruction text. Participants were told that they would have to type words from a sequence of letters using their eyes subsequently.

**Figure 1:**
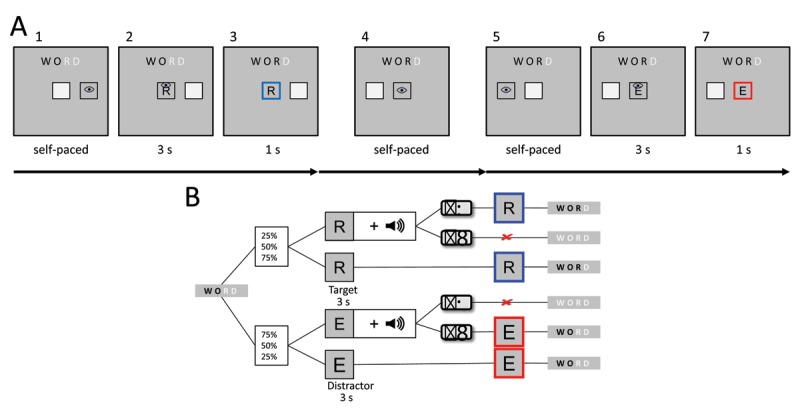
**A:** Two subsequent trials, once for a target (1–3), once a distractor letter (4–7). **A1:** Representative depiction of the presented screen before starting a trial. **A2:** After first gazing into the outer activation box that was randomly presented either to the left or right of the central box, participants had to make a saccade to the center to start a trial. A highlight fed back whether gaze position was registered inside the respective box. The word presented above indicated which letter was correct, which was always the leftmost letter given in gray (here: *R*). **A3:** After 3 s of keeping the gaze inside the central box and handling a potential GoNoGo signal correctly (after tone prompt in 50% of trials: press left key for distractors and right key for targets), a highlight fed back that the trial had been finished. **A5–A7:** Next trial, in this case with a distractor letter that has been handled correctly. B: Flowchart of trials.

Participants saw a gray screen with a light gray quadratic box with black edges with a length of 4.5 degree visual angle presented in the screen center in addition to a similarly shaped and sized box right or left to the central box. Above, in the upper horizontal center, a four letter neutral word was presented in black and light gray letters (Figure 1 A1). Whenever participant’s gaze position was registered inside a box, the box changed color to the background gray as a feedback. To start a trial, participants first had to fixate on the outer box and saccade to the center and remain inside the central box with their gaze. Upon entering the box, a letter was presented that either matched (= target) the leftmost light gray letter in the word presented above or mismatched this letter (= distractor). Whether a target or a distractor letter was presented was determined for each trial depending on the level of stimulus probability in the block (25%, 50%, 75%). In 50% of trials, a 200 ms 440 Hz sinewave tone prompted participants to indicate via key press whether the central letter was matching the letter presented above (= Go; target: right arrow key, distractor: left arrow key). Hence, the probability of a tone to be presented was independent from the probability of a presented letter to be a target. Participants now had to keep their gaze for 3 s inside the central box. If they had been prompted via tone before, participants were to press the respective correct key in the same time (Figure 1 A2). After 3 s a highlight signalled to participants that the trial had been handled correctly (1 s, blue frame for target, red frame for distractors; Figure 1 A3). Participants could now rest their eyes (Figure 1 A4) and subsequently start the next trial (Figure 1 A5–A7) until they had completed all letters. Once a word was finished, a new neutral four letter word was presented. Participants were instructed that the experiment would end, once all words were completed. Whenever participants pressed a key unprompted, did not press a key when prompted, or pressed an incorrect key, they were punished by having to retype the last two letters in the above presented word, thus prolonging the experiment. When participants blinked longer than 200 ms or left the central box with their gaze position, a new trial had to be started. Figure 1 C visualizes all possible outcomes as a flowchart.

In total, participants had to write 10 words à four letters per block, which equals at least 120 target letters in total, when no errors were made. Each block ended after 10 words had been presented and was followed by a short pause and an instruction screen that announced the subsequent stimulus probability level. The experiment itself lasted between 25 and 35 minutes.

#### Data analysis

Gaze data were saved using SMI iViewX. Subsequently, SMI event detector was employed to differentiate fixations from saccades and blinks. All trials with cumulated blink duration longer than 200 ms were excluded from further analyses. Blinks shorter than 200 ms were interpolated using a modified algorithm based on Georgi, Kowalski, Ehlers, and Huckauf (2014). This procedure was chosen instead of treating blinks as missing data to preclude potential artifacts resulting from differential blink frequencies across experimental conditions. For each trial, the average pupil diameter of the first 20 ms of gazing at the central box was taken as a local baseline. This average was then subtracted from the respective following trial. Subsequently, data were downsampled to 50 Hz for all following analyses.

Reaction times were analyzed for correct answers only. Reaction times may be distorted by outliers (Ratcliff, 1993), which is why reaction times deviating more than two standard deviations from average reaction times per condition per participant were removed (2-SD trimming). All data were aggregated and statistically analyzed using scripts in Python and R (R-Core-Team et al., 2013). For Bayesian analyses JASP was used (JASP-Team et al., 2018).

### Results

#### Pupil diameter

**Figure 2:**
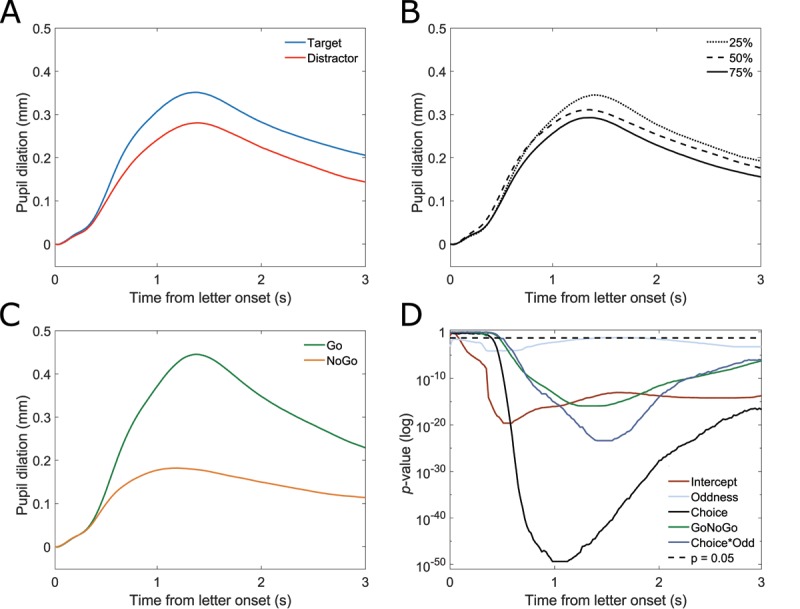
A–C: Pupil dilation over time against local baseline. A: target (blue) and distractor (red). **B:** stimulus probabilities: 25% (dotted), 50% (dashed), and 75% (solid). **C:** Go (green) and NoGo (yellow). **D:** *p*-values for a functional linear mixed model over time. Choice: dark grey, stimulus probability (oddness): light blue, GoNoGo: green, choice*stimulus probability: dark blue, intercept: light brown, α = 0.05: dashed black. Based on n = 44 participants and n = 13551 individual trials.

Pupil dilated relative to the local baseline over time in all conditions. Replicating effects of movement (Richer & Beatty, 1985), Go trials elicited a stronger pupil dilation than NoGo trials (Figure 2C). Assessing the statistical significance of the remaining factors, a within ANOVA was fitted for the average pupil diameter during letter presentation. Significant effects were found for decision making, stimulus probability and their interaction. Decision making affected pupil dilation (*F*(1,43) = 121.697, *p* < 0.001, \eta _p^2 = 0.731), with an on average larger dilation for target compared to distractor letters (Figure 2A). Stimulus probability also affected pupil diameter, albeit by a smaller effect size (*F*(2,86) = 24.238, *p* < 0.001, \eta _p^2 = 0.360). Here, pupil dilated more for rare stimuli compared to those that were presented at equal probability and frequently in a graduated manner (Figure 2B). Moreover, the interaction of decision making and stimulus probability was found significant (*F*(2,86) = 10.626, *p* < 0.001, \eta _p^2 = 0.198).

**Figure 3:**
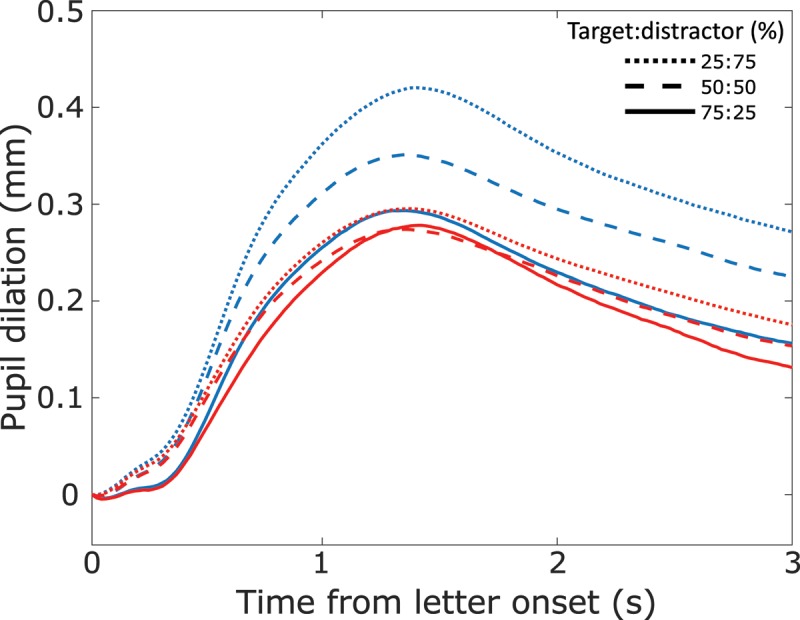
Pupil dilation for target (blue) and distractor (red) along target:distractor ratio. Dotted lines: 25% targets, dashed lines: 50% targets, solid lines: 75% targets.

Examining the statistical meaningfulness of each factor over time and further considering the nested data structure, a linear mixed model (LMM) was fitted functionally (trials nested in participants and conditions). Figure 2D visualizes the *p*-values of effects as calculated in the functional LMM of each factor and interaction over time, with smaller values indicating higher statistical significance. A False-Discovery-Rate correction was used to correct for the large number of tests using the Benjamini-Hochberg procedure (Benjamini & Hochberg, 1995), the similar method was later also used for a functional model in Experiment 2. Partially very low *p*-values should not be over interpreted, but interpreted as being beyond a threshold of *p* < 0.001. Still, they reveal information about the time course of the effects on pupil diameter, e.g. decision making affected pupil dilation significantly before GoNoGo. When also considering GoNoGo as a factor and the nested data structure, decision making/choice and the interaction reveal clear effects, whereas the main effect of stimulus probability is drastically smaller and partially at the exact threshold for statistical significance (here: α = 0.05; roughly between 1.1 s and 2 s after letter onset). Pupil differentiated choice 120 ms earlier than stimulus probability. This sequence is also evident in descriptive pupil sizes (see Figures 2A–B and 3). The employed model was derived from the full model using AIC-based backward selection; this also implies that effects in Go and NoGo conditions were not different, as no interaction was found.

In Figure 3, pupil diameter is plotted for target and distractor conditions for all three blocks: in all blocks, pupil dilated more for target than distractor letters. When targets were rare, this difference was maximal; but even when targets were more frequent than distractors, pupil dilated more for targets than for distractors. Furthermore, it is discernible that stimulus probability mainly affected pupil dilation for target letters, whereas pupil signal courses for distractor letters stayed relatively similar throughout all blocks.

#### Reaction times

**Figure 4:**
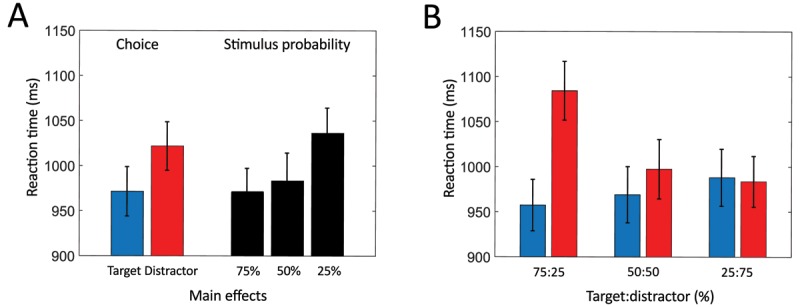
Reaction times. **A:** main effects for choice (target = blue, distractor = red) and stimulus probability (black). **B:** Reaction times for target and distractor along target:distractor ratio. Error bars indicate standard errors of the mean.

Analyzing reaction times statistically, a within 2 × 3 ANOVA was fitted for the effects of choosing, stimulus probability, and their interaction. Effects were found for both decision making (*F*(1,43) = 26.939, *p* < 0.001, \eta _p^2 = 0.385), with participants responding faster to targets than to distractors (Figure 4A left) and stimulus probability (*F*(2,86) = 7.655, *p* < 0.01, \eta _p^2 = 0.151), with participants responding faster for frequent than for rare letters (Figure 4A right). The interaction of decision making and stimulus probability did not reach statistical significance (*F*(2,86) = 2.353, *p* = 0.122, \eta _p^2 = 0.052). Figure 4B visualizes the additive effects of decision making and stimulus probability. Participants were generally highly accurate in pressing the correct key in Go-trials (*M* = 97.36%, *SD* = 2.55 %).

### Discussion

In Experiment 1, the effects of decision making (target vs distractor letters) were conjointly investigated with varying stimulus probabilities of these letters in a blockwise manner. As a replication of previous results, it was found that pupil dilates more for target than distractor letters at even probabilities of presentation (Strauch et al., 2018). This finding is in line with EEG-investigations, indicating that deciding on stimuli causes activity in the LC and the release of norepinephrine (Nieuwenhuis, Aston-Jones, & Cohen, 2005). Examining the possible interaction of these effects with stimulus probability in pupil dilation, targets were either presented at a likelihood of 25%, 50% or 75%. As in previous works, pupil dilated more for rare compared to frequent stimuli (Murphy, O’Connell, et al., 2014; Nieuwenhuis et al., 2011), similar to effects described for the P3 (Duncan-Johnson & Donchin, 1977; Verleger, Grauhan, & Smigasiewicz, 2016). However, targets always elicited a stronger dilation than distractors, even when they were frequent and distractors rare. This result is in line with previous EEG research (Bruin & Wijers, 2002). Extending existing oddball investigations using pupillometry (Murphy, O’Connell, et al., 2014; Nieuwenhuis et al., 2011), the oddball effect was also visible independent from a key press in the NoGo condition, excluding effects to result from motor execution alone. Here presented findings for pupil dilation reflect existing P3 research, supporting the notion of a high comparability of effects between both measures (Nieuwenhuis et al., 2011).

An ANOVA revealed significant main effects for choice, as well as probability and a significant interaction of both factors. Further examining the relationship of these factors onto pupil dilation, a linear mixed model was fitted, demonstrating the same effects to be statistically significant. However, the main effect of stimulus probability was substantially reduced and very weak compared to the other effects when considering the nested data structure. Targets, but not distractors, were mostly affected by stimulus probability. This raises the question, whether stimulus probability alone has any effect on pupil dilation, arguing for an experimental investigation into this question.

How did targets differ from distractors and why did they cause a larger pupil dilation? Findings argue for the role of relevance for effects to emerge, as proposed previously for a number of effects in pupillometry (Nieuwenhuis et al., 2011). In an oddball task, Verleger, Keppeler, Sassenhagen, and Śmigasiewicz (2018) added a second dimension to their stimuli (colored letters) and varied feature-response mappings. In line with the idea of relevance as necessary factor, the oddball effect was only obtained when participants knew which dimension was relevant and what response was required. Decision making in our experiment could thus mean deciding on a stimulus relevance: target letters were goal relevant (in a way that only they allowed finishing the experiment) whereas distractor letters were only task but not goal relevant (they had been attended to, to answer potential Go prompts, but did not allow shortening the experiment).

Besides pupillometric data, reaction times were analyzed. An ANOVA showed a significant main effect for choice and stimulus probability, but not for their interaction. Reaction times were demonstrated to be faster for targets than for distractors, replicating a number of investigations (De Gee et al., 2014; Wolfe, Palmer, & Horowitz, 2010, e.g.). In line with previous findings (Gordon, 1967, e.g.), participants reacted faster to frequent than to rare stimuli. Descriptively, reaction time was altered stronger for distractor than for target letters. Interestingly, especially rare stimuli were differing from equiprobable and frequent stimuli, which could be due to a ceiling/floor effect. Shorter reaction times are connected to a higher expectancy to stimuli (Gordon, 1967). Hence, stimulus probability and choice modulated expectancy regarding letters. Targets have been generally expected more than distractor letters, which could argue for a matching process of pre-activated inner targets with incoming stimuli underlying the ‘yes’/‘no’ choice in this experiment.

## Experiment 2

Clarifying experimentally what level of stimulus relevance drives the interaction with stimulus probability, Experiment 2 was conducted. Here, we tested stimuli that were irrelevant to task and goals, stimuli that were relevant to task but not goals, and stimuli relevant to both task and goals. Further, it was investigated whether stimulus probability alone affects pupil dilation and reaction times besides attempting to replicate findings of Experiment 1 in conditions similar for both Experiments. Moreover, results of both experiments should contribute to the development of a framework that may predict pupil dilation while processing a given stimulus.

### Methods

Experiment 2 replicated Experiment 1 except for the following changes:

#### Participants

Twenty-five participants (*M*_Age_ = 23.4 years, 17 female) with normal or corrected to normal vision took part in Experiment 2. Data could be analyzed for all participants.

#### Design and task

**Figure 5:**
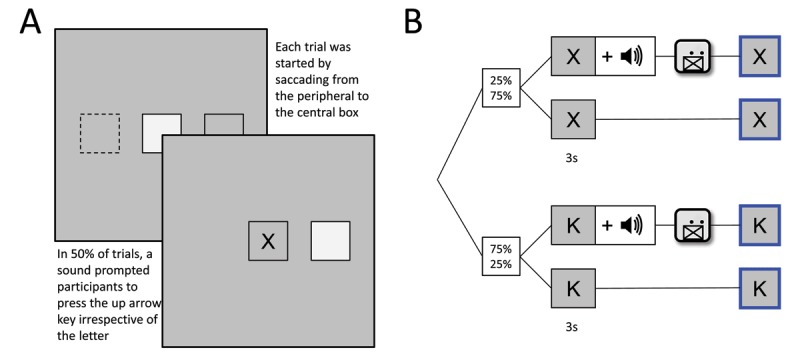
A: Trials were performed as in Experiment 1, however, no target word was presented during Block 1, letters were therefore neither targets nor distractors. **B:** Flowchart of trials: at a chance of either 25% or 75% the letters X or K were presented upon fixation of the central box. In 50% of cases, a sinewave tone prompted participants to press the up key; participants needed to keep gazing into the central box for 3 s. Else, no key press was required until a highlight fed back that the trial had been handled correctly.

A within design was employed, with three blocks presented to participants. In the first block, participants performed a comparable task as in Experiment 1, however, no word was presented in the upper center of the screen. At a chance of 25%, (75%), the letter K was presented, whereas in the remaining 75% (25%) the letter X was presented when a trial started. Whether X or K was the frequent (75%) or rare (25%) stimulus throughout the first block was determined by chance at the beginning of the block. Hence, stimulus relevance was not manipulated in Block 1 (see Figure 5). As in Experiment 1, participants had to react to a Go signal in 50% of trials via key press, here they always had to press the up key irrespective of the presented letter. Participants were instructed to press the key upon Go signal as fast as possible. Correctly handled trials were fed back using a 1 s lasting feedback (thicker blue frame around letter). In total, participants needed to select at least 40 letters. If a key was pressed unprompted or a key press was missed, two more trials were added. The following two blocks were an exact replication of the 25:75 target:distractor and 75:25 target:distractor blocks of Experiment 1. The sequence of these two blocks was determined by chance. This further allowed investigating the role of relevant compared to irrelevant stimuli in the interaction with stimulus probability more closely. Adding Block 1, three levels of stimulus relevance result: *letter in Block 1* (irrelevant to task and goals), *distractor in Block 2 and 3* (relevant to task, but not goals), and *target in Block 2 and 3* (relevant to task and goals).

### Results

#### Pupil diameter

**Figure 6:**
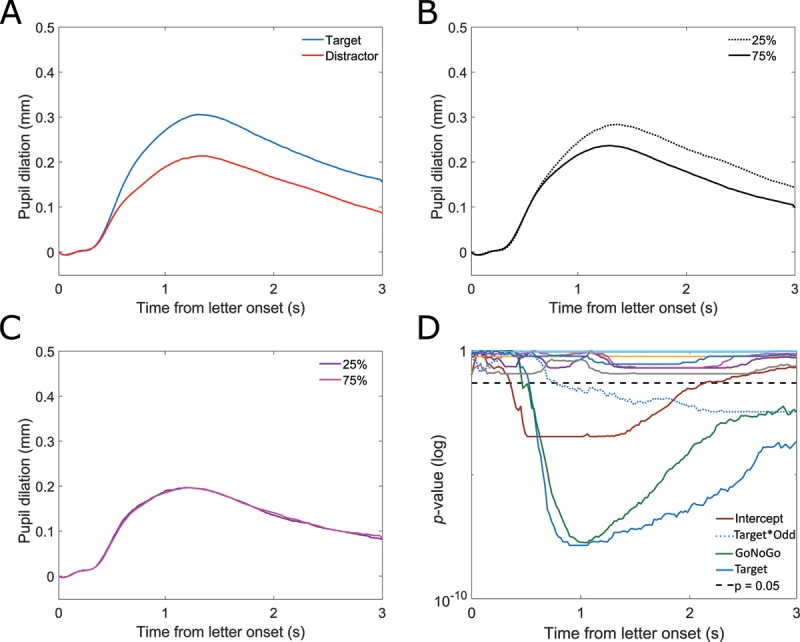
A–C: Pupil dilation against local baseline. **A:** target (blue) and distractor (red). **B:** stimulus frequencies: 25% (dotted) and 75% (solid) during blocks 2 and 3. **C:** stimulus frequencies: 25% and 75% during Block 1. **D:** *p*-values for a functional linear mixed model over time using the factors stimulus relevance (Block 1, distractor, target), GoNoGo, and 25%75%, as well as their interactions. Other factors and factor levels, including stimulus probability and distractor, were non significant. Target: blue, GoNoGo: green, target*stimulus probability: dotted blue, intercept: light brown, α = 0.05: dashed black. Based on n = 25 participants and n = 7052 individual trials.

As in Experiment 1, a within ANOVA was fitted for the factors stimulus relevance and probability. Replicating Experiment 1, stimulus relevance, stimulus probability and their interaction showed significant effects. Pupil again dilated stronger for target compared to distractor letters (*F*(1,24) = 41.014, *p* < 0.001, \eta _p^2 = 0.631; Figure 6A) and for rare compared to frequent letters in blocks 2 and 3 during which the decision task had to be performed conjointly (*F*(1,24) = 20.213, *p* < 0.001, \eta _p^2 = 0.457; Figure 6B). The interaction of decision making and stimulus probability was significant (*F*(1,24) = 20.657, *p* < 0.001, \eta _p^2 = 0.463). In Block 1, where stimuli were neither targets nor distractors however, pupil diameter showed an on average almost identical dilation for rare and for frequent stimuli (Figure 6C). A paired samples t-test for the average pupil dilation during letter presentation also shows no significant difference between rare and frequent stimuli (*t*(24) = 0.031, *p* = 0.976); a Bayesian paired samples t-test indicates a BF_01_ = 4.741 times higher chance for the data under the null compared to the alternative hypothesis. Testing whether distractors in blocks 2 and 3 elicited differential pupil dilations from letters in Block 1, a t-test for paired samples and a Bayesian t-test for paired samples were performed. The t-test suggests *t*(24) = 0.419 no significant difference; chances for the data under the null hypothesis were found BF_01_ = 3.493 times higher than under the alternative hypothesis. This indicates that irrelevant and task relevant but not goal relevant stimuli elicit similar pupil size changes.

**Figure 7:**
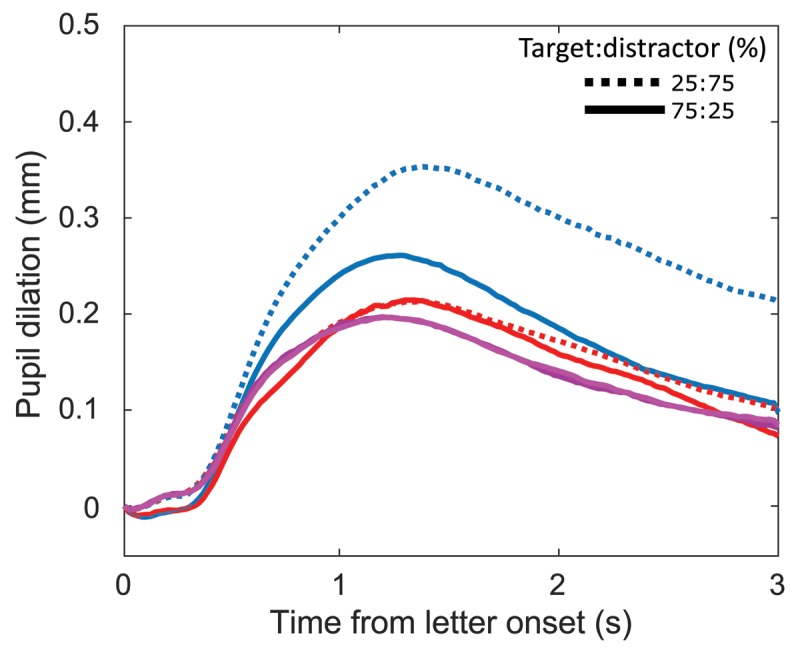
Pupil dilation for target (blue), distractor (red), as well as for rare (purple) and frequent (pink) letters during Block 1. Pupil dilation for target and distractor conditions split along target:distractor ratio. Dotted lines: 25% targets, solid lines: 75% targets.

Similar to Experiment 1, a LMM was fitted (Figure 6D). While the fitted model differed from the model for Experiment 1 in that it also included the three-way interaction of stimulus relevance, strimulus probability, and GoNoGo from the model in Experiment 2, the temporal dynamics of the principal effects are comparable. A significant main effect was found for stimulus relevance (task and goal irrelevant, task but not goal relevant, task and goal relevant), more precisely, target letters were different from distractor letters and letters in Block 1 while the latter two did not differ. That is, the interaction of stimulus relevance and stimulus probability, as well as the main effect for stimulus relevance was driven solely by stimuli that were goal-relevant to participants. Moreover, an interaction of target letters with stimulus probability was found, but no main effect of stimulus probability. Pupil size was again earlier (240 ms) significantly affected by targets than by the interaction of targets with stimulus probability, which is also visible in average pupil sizes (see Figures 6A–B and 7). As in Experiment 1, GoNoGo had a significant impact on pupil dilation (Figure 6D).

Figure 7 visualizes descriptive pupil dilation for target and distractor letters as well as the letters presented in Block 1. As in Experiment 1, pupil dilated strongest when targets were rare, followed by targets when frequent. Distractor letters were almost identically affecting pupil diameter irrespective of stimulus probability. Pupil dilated descriptively slightly more for distractors than for the letters presented in Block 1, starting at about 1.25 s after letter onset.

#### Reaction times

**Figure 8:**
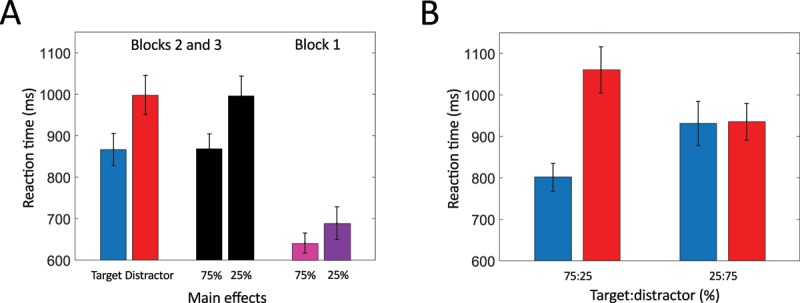
Reaction times. **A:** effects for stimulus relevance (target: blue, distractor: red, irrelevant frequent: pink, irrelevant rare: purple) and stimulus probability (black). **B:** Reaction time for target and distractor along target:distractor ratio. Error bars indicate standard errors of the mean.

A 2 × 2 within ANOVA was fitted to compare reaction times for the remaining two blocks, which replicated findings from Experiment 1. Stimulus relevance affected reaction times significantly (*F*(1,24) = 28.643, *p* < 0.001, \eta _p^2 = 0.544; correct reactions were faster for target compared to distractor trials (Figure 8A left). Stimulus probability similarly affected reaction times differentially (*F*(1,24) = 38.886, *p* < 0.001, \eta _p^2 = 0.618), with faster reactions for frequent than for rare stimuli (Figure 8A middle). No interaction of choice and probability was found (*F*(1,24) = 0.004, *p* = 0.950). The interplay of relevance and stimulus probability is visualized in Figure 8B. Participants were generally faster to respond to the Go prompt in Block 1 (right), during which no additional task was given, than in blocks 2 and 3. Differences in reaction times for rare compared to frequent letters during Block 1 were found to be non significant using a paired samples t-test (*t*(24) = 2.020, *p* = 0.055; Figure 8A right). Testing whether reaction times for rare and frequent stimuli are equal, a Bayesian paired samples t-test was performed. Chances are BF_01_ = 0.837 times higher for the data under the null hypothesis compared to the alternative hypothesis. As in Experiment 1, participants were highly accurate in their responses (*M* = 98.79%, *SD* = 2.00%).

### Discussion

Investigating experimentally whether stimulus probability would affect pupil diameter and reaction times without assigned relevance, Experiment 2 was conducted. Moreover, levels of relevance were compared and the principal effects of Experiment 1 were tested for replicability.

In Block 1 of Experiment 2, stimulus probability was the only factor. Neither pupil nor reaction times distinguished the stimuli that differed only in their probability, although a descriptive tendency towards faster reactions for frequent stimuli was observed. In contrast to Qiyuan et al. (1985), but in line with most other existing research, stimulus relevance was thus necessary for oddball effects to emerge.

Experiment 1 suggested a crucial role of relevance for effects in pupil dilation. Understanding what specific type of relevance is underlying effects, three different levels of relevance were tested: irrelevant to task and goals (Block 1), relevant to task but not goals (distractors), and relevant to task and goals (targets). For participants, it was irrelevant whether presented letters in Block 1 were rare or frequent, as both required a similar key press and were associated with identical consequences. Distractors may be considered as irrelevant to the goal of the participant: proceeding with writing to finish the experiment, but must be considered as task relevant, since an eventual necessary key response had to be handled correctly. Targets were task relevant for the same reason, but also goal relevant, given that only they allowed to write words and thus end the experiment. Fitting a linear mixed model for pupil diameter further revealed that only target stimuli had a main effect and interacted with stimulus probability, whereas no effect or interaction was found for stimuli without assigned relevance or distractor stimuli. Hence, these results argue for goal relevance instead of just task relevance as precondition for oddball effects. For a stimulus to be goal relevant, a motor execution was not strictly necessary: Effects emerged in the NoGo condition as well and thus under inhibition (Kiefer, Marzinzik, Weisbrod, Scherg, & Spitzer, 1998). This is in line with earlier findings arguing for goal (= motivational) rather than simply task relevance Simpson (1969).

Most findings of Experiment 1 were clearly replicated. In Experiment 1, the main effect of stimulus probability was drastically reduced when considering the nested data structure, in Experiment 2 however, no significant main effect of stimulus probability could be found in the LMM. Taken together, this argues for separate effects of stimulus relevance and stimulus probability, however, stimulus relevance may be considered a precondition for the effect of stimulus probability.

The comparison of pupil dilation and reaction times for the interaction of choice and stimulus probability reveals an on average inverse relation in both experiments: For pupil dilation, targets evoked the largest dilation and were affected by stimulus probability, whereas irrelevant stimuli and distractors remained unaffected. For reaction times however, stimulus probability mostly affected distractors with rare distractors evoking the longest reaction time, whilst targets remained largely unaffected, especially in Experiment 1. Effects may be explained for both measures individually based on the literature. Still, it remains an open question why differences in pupil size, and thus activation, seem not associated with differential reaction times on average.

## General Discussion

Employing a sequential letter selection ‘yes’/’no’ paradigm, the influence of decision making and stimulus probability on pupil dilation was assessed in two experiments. We found stimulus relevance to be a precondition for effects of stimulus probability, but not vice versa. Different theoretical models have been proposed to explain the effects of decision making and stimulus probability comprehensively. In the following paragraphs, we shortly describe the most prominent theories and discuss them with regard to their capability of explaining the here presented results, first for decision making and second for the assumed causes of the oddball effect.

### Embedding findings in theories of decision making and stimulus probability

For choosing between two options, the **drift diffusion model** represents the most prominent account: Evidence is assumed to be accumulated for both alternatives simultaneously over time (*drift rate*). A decision is reached, as soon as evidence for one option surpasses a *threshold* (Ratcliff, Smith, Brown, & McKoon, 2016). As the accumulation process is stochastic in nature, noise may cross the threshold and thus lead to incorrect decisions. The higher the threshold, the longer the reaction time and the earlier and more accumulated evidence, the shorter the reaction time. Regarding pupil size, results have indicated both, evidence in favour of a larger pupil dilation representing a higher threshold (Cavanagh, Wiecki, Kochar, & Frank, 2014) and larger pupil dilation representing more accumulated evidence (De Gee et al., 2014; Murphy, Vandekerckhove, & Nieuwenhuis, 2014). The results of decision making and stimulus probability in pupil diameter and reaction times represent challenges to both notions.

Assuming that pupil size represents the threshold, here reported pupil dilation would indicate differential thresholds for target letters, but not distractor letters due to a variation in stimulus probability. However, reaction times differed not only for target, but also for distractor letters. Pupil dilation would further suggest faster responses to distractors than to targets, since a lower threshold should be connected to faster reaction times, however, the opposite pattern is found. Though, it may not be excluded that thresholds were collapsing after pupil dilation peaked at about 1.5 s after letter onset. Thresholds for ‘yes’ (targets) and ‘no’ (distractors) might have collapsed differentially: if not enough evidence had been accumulated for ‘yes’ by a certain time, a no-decision was performed. The time when thresholds collapsed could then have been affected by stimulus probability and thus expectancy. This could include a mechanism during which ‘no’ is only selected, if the evidence for ‘yes’ was not sufficient, an idea that has also received some backing from the activation of neural correlates specific for ‘yes’ responses (Aston-Jones & Cohen, 2005; Deco, Pérez-Sanagustín, De Lafuente, & Romo, 2007).

Assuming that pupil size represents the evidence accumulation process, larger pupil sizes should go in hand with faster reaction times. This assumption may be supported by the finding that target letters elicited a larger dilation than distractor letters and were faster responded to. Still, reaction times were not shorter with further increasing dilation with rare targets compared to equiprobable targets, which would argue for a ceiling effect. The model seems to need further specification to fully account for the here presented results of pupil dilation and reaction times. Moreover, if pupil dilation was increasing due to accumulating evidence leading to a decision, it is hard to explain why pupil size was sustained larger for targets than distractors also considerably after a decision had been reached, that is, after about 2 s of letter presentation.

Sustained higher activation for target letters might be interpreted in favor of the idea of pupil dilation reflecting **response facilitation** (Nieuwenhuis et al., 2005). Our data support this notion: larger activation for goal relevant stimuli (targets) is associated also with shorter reaction times. This facilitation however only affected target and thus goal relevant stimuli, not distractors. Furthermore, even after pressing a key, pupil remained larger for target compared to distractor letters, which might indicate that activation remains higher as long as the relevant stimulus is present in ones mind for potentially further upcoming responses or mental operations.

Based on the theory of event coding (Hommel, Müsseler, Aschersleben, & Prinz, 2001), Verleger et al. (2018); Verleger and Śmigasiewicz (2016) propose that the **pairing of stimuli with responses** creates event files (S-R files), that are differentially activated for rare and frequent stimuli-response pairs. Whenever a rare stimulus-response file needs to be reactivated, this goes in hand with longer reaction times and a stronger increase in activation than for a frequent S-R file. The observed oddball effects are principally in line with this idea, however, the S-R link hypothesis may not explain differences between target and distractor, that are also found under equiprobability.

In both experiments, differences in **goal relevance** (i.e., target vs. distractor) were the precondition for effects to emerge, shaping it the more parsimonous explanation. However, this does not exclude e.g. response facilitation or S-R links to affect pupil dilation and reaction times in a later step in the interaction with stimulus probability.

Summing up, different theories may account for different parts of the results found investigating decision making and stimulus probability. In what follows, a framework is described that is able to explain the here reported differences in pupil size comprehensively.

### Integrating framework to effects of stimulus relevance and probability

Comparing the pupillary signals for the different levels of stimulus relevance and stimulus probability reveals an earlier effect of stimulus relevance than stimulus probability on pupil dilation. In both experiments, pupil dissociated about 200 ms earlier for stimulus relevance than for stimulus probability (Experiment 1:120 ms, Experiment 2:240 ms). This pattern is also evident when it comes to the statistical significance of the main effect of stimulus relevance and the interaction of relevance with probability, reaching significance and peaking earlier for relevance than for the interaction of relevance and probability in the functional linear mixed models for Experiment 1 and Experiment 2. The chronology of effects together with the missing main effect of stimulus probability in the experimental test for it in Experiment 2 argue for a sequential processing of stimulus relevance and probability: First, a decision is reached regarding a stimulus’ goal relevance and, only if relevant, it is further evaluated. Second, stimulus probability is assessed regarding the stimulus’ value, potentially based on an expectancy-value mechanism. This mechanism is reflected also in pupil diameter: Irrelevant stimuli elicit the smallest pupil dilation, whereas relevant stimuli elicit a stronger pupil dilation early on. The higher the relative value of a relevant stimulus, the more pupil dilates subsequently. In the here reported experiments, the subjective value to participants may be assumed to be higher to participants for rare than for frequent target stimuli, resulting in the largest pupil dilation for goal relevant rare stimuli.

**Figure 9:**
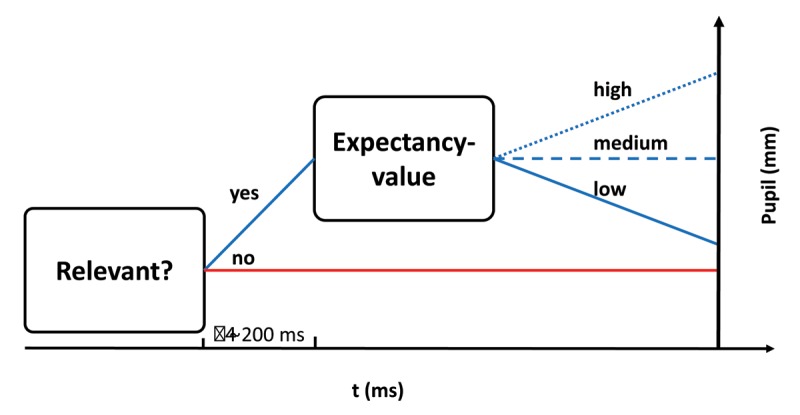
Proposed framework for integrating effects of stimulus relevance and stimulus probability and predicting pupil size when processing stimuli. Pupil dissociates earlier for relevance than for stimulus probability, arguing for separate processes. Given that a stimulus is relevant, pupil dilates differentially based on an expectancy-value mechanism.

Figure 9 visualizes this framework for integrating effects of stimulus relevance and value-expectancy on the basis of pupil dilation. While the first step could comprise models on decision making, such as the drift diffusion model with pupil diameter representing evidence accumulation and/or (a) collapsing threshold(s), the second step could include, but would not be limited to theoretical explanations for the oddball effect, such as response facilitation. Data from the two experiments presented here point to an offset of about 200 ms between the effects of stimulus relevance and further evaluation coming into place; however, it is conceivable that more complex expectancy-value considerations would enlarge this offset. Hence, the precise length of the offset between the two processes affecting pupil dilation needs further investigation. Possible ways to falsify this model might include testing the model with EEG for chronology and different stratification of signals, as well as manipulating perceived value to participants. One possibility would be to manipulate the consequences of certain stimuli by assigning more or less reward or punishment to them, which should affect pupil dilation (Varazzani, San-Galli, Gilardeau, & Bouret, 2015, e.g.). It has to be noted that at least in Experiment 1, signal courses for distractors were slightly, albeit non-significantly, different between the blocks: Rare distractors tended to dilate pupil more than frequent distractors. Besides the here proposed framework, effects of novelty and surprise are known to affect pupil dilation (Nieuwenhuis et al., 2011; Preuschoff, ’t Hart, & Einhäuser, 2011), which might account for this non-significant finding.

### Future research and limitations

In the here presented experiments, pupil dilation has again proven to constitute a viable indicator of ongoing cognition. Pupil size reveals whether a stimulus is relevant or not, further, in a second step it indicates how big the subjective value of such a stimulus is to an observers’ goals in comparison to other stimuli. Potential applications range from the possibility to read an opponent’s intention above chance (Naber, Stoll, Einhäuser, & Carter, 2013, e.g.) or enhancing pupil-adaptive human-machine-interfaces (Strauch, Ehlers, & Huckauf, 2017). Of course, results reported in this article stem from strictly controlled laboratory experiments, which makes retrieving such information substantially easier than in the wild.

While no significant main effect of stimulus probability was found when testing for it experimentally in Block 1 of Experiment 2, this does not rule out that an oddball effect might be found if rare stimuli were substantially rarer, as the oddball effect is stronger the rarer the rare stimulus is (Duncan-Johnson & Donchin, 1977). Given the current overlap of curves for rare and frequent stimuli, however, any stimulus must be drastically rarer than in this investigation for a potential effect.

Reported reaction times have to be treated with caution when comparing results with most existing literature. That is, the task employed in the here presented experiments differs in that participants had a comparably long time to answer via key press (3 s). Due to the strong consequence of an incorrect response, participants were very accurate in their responses. Therefore, it may be assumed that speed has been traded in for high accuracy. Furthermore, reaction times were assessed only in one half of the trials and therefore do not allow similar multilevel analyses as for pupil dilation. In future investigations, the NoGo condition could thus be dropped, as no interaction with the effects of either decision making or stimulus probability was found in both experiments.

Pupil dilated more for Go than for NoGo trials. Here, it has to be noted that (unexpected) tones, such as the sound prompt used here, have likely contributed to the observed effects (Zekveld, Koelewijn, & Kramer, 2018). In a previous investigation using a highly comparable design and a similar tone, effects isolable to the tone alone were shown to be about one tenth of the absolute GoNoGo effect (Strauch et al., 2018). Hence, we maintain the assumption that the effect of the tone is rather small in comparison to the other effects (i.e. deciding to press a key and the key press).

Signaling decision making finely grained, pupil diameter may help discerning ongoing cognition and behavior. Coming back to the example from the introduction, what would pupil dilation tell us about a specific bus arriving in a series of buses of different lines at a bus stop? Based on the framework visualized in Figure 9, pupil should dilate more for buses going to our destination (= relevant buses), than for buses going elsewhere. If several buses would go to our destination but would take shorter or longer, pupil should dilate more for the fastest possibility, as expected value should be highest to the commuter.

### Conclusion

In two experiments, we investigated the effects of decision making and stimulus probability on pupil dilation. A presented stimulus’ relevance and the probability of a stimulus both affected pupil diameter and reaction times. However, for pupil dilation, analyses strongly suggest that oddball effects only emerged when stimuli were goal relevant. This points to a mechanism during which stimuli are first classified regarding their goal relevance and, in a temporarily subsequent second step, assessed regarding their goal relevant value.

## Data Accessibility Statement

Data from the experiments as well as the scripts for experiments, signal processing and statistical analyses may be retrieved from the open science framework via https://osf.io/yfszq/.

## References

[1] Aston-Jones, G., & Cohen, J. D. (2005). An integrative theory of locus coeruleus-norepinephrine function: adaptive gain and optimal performance. Annual Review of Neuroscience, 28, 403–450. DOI: 10.1146/annurev.neuro.28.061604.135709 16022602

[2] Beatty, J., & Wagoner, B. L. (1978). Pupillometric signs of brain activation vary with level of cognitive processing. Science, 199(4334), 1216–1218. DOI: 10.1126/science.628837 628837

[3] Benjamini, Y., & Hochberg, Y. (1995). Controlling the false discovery rate: a practical and powerful approach to multiple testing. Journal of the Royal Statistical Society. Series B (Methodological), 289–300. DOI: 10.1111/j.2517-6161.1995.tb02031.x

[4] Bruin, K., & Wijers, A. (2002). Inhibition, response mode, and stimulus probability: a comparative event-related potential study. Clinical Neurophysiology, 113(7), 1172–1182. DOI: 10.1016/S1388-2457(02)00141-4 12088714

[5] Cavanagh, J. F., Wiecki, T. V., Kochar, A., & Frank, M. J. (2014). Eye tracking and pupillometry are indicators of dissociable latent decision processes. Journal of Experimental Psychology: General, 143(4), 1476 DOI: 10.1037/a0035813 24548281 PMC4114997

[6] Deco, G., Pérez-Sanagustín, M., De Lafuente, V., & Romo, R. (2007). Perceptual detection as a dynamical bistability phenomenon: a neurocomputational correlate of sensation. Proceedings of the National Academy of Sciences, 104(50), 20073–20077. DOI: 10.1073/pnas.0709794104 PMC2148424 18077434

[7] De Gee, J. W., Colizoli, O., Kloosterman, N. A., Knapen, T., Nieuwenhuis, S., & Donner, T. H. (2017). Dynamic modulation of decision biases by brainstem arousal systems. Elife, 6, 1–36. DOI: 10.7554/eLife.23232 PMC5409827 28383284

[8] De Gee, J. W., Knapen, T., & Donner, T. H. (2014). Decision-related pupil dilation reflects upcoming choice and individual bias. Proceedings of the National Academy of Sciences of the United States of America, 111(5), E618–E625. DOI: 10.1073/pnas.1317557111 24449874 PMC3918830

[9] Duncan-Johnson, C. C., & Donchin, E. (1977). On quantifying surprise: The variation of event-related potentials with subjective probability. Psychophysiology, 14(5), 456–467. DOI: 10.1111/j.1469-8986.1977.tb01312.x 905483

[10] Ehlers, J., Strauch, C., Georgi, J., & Huckauf, A. (2016). Pupil size changes as an active information channel for biofeedback applications. Applied Psychophysiology and Biofeedback, 41(3), 331–339. DOI: 10.1007/s10484-016-9335-z 27113096

[11] Einhäuser, W. (2017). The pupil as marker of cognitive processes In Computational and cognitive neuroscience of vision (pp. 141–169). Springer DOI: 10.1007/978-981-10-0213-7_7

[12] Einhäuser, W., Koch, C., & Carter, O. L. (2010). Pupil dilation betrays the timing of decisions. Frontiers in Human Neuroscience, 4, 1–9. DOI: 10.3389/fnhum.2010.00018 20204145 PMC2831633

[13] Georgi, J., Kowalski, D., Ehlers, J., & Huckauf, A. (2014). Real-time feedback towards voluntary pupil control in human-computer interaction: Enabling continuous pupillary feedback. In International workshop on icts for improving patients rehabilitation research techniques (pp. 104–114). DOI: 10.1007/978-3-662-48645-0_10

[14] Gilzenrat, M. S., Nieuwenhuis, S., Jepma, M., & Cohen, J. D. (2010). Pupil diameter tracks changes in control state predicted by the adaptive gain theory of locus coeruleus function. Cognitive, Affective, & Behavioral Neuroscience, 10(2), 252–269. DOI: 10.3758/CABN.10.2.252 PMC3403821 20498349

[15] Gordon, I. (1967). Stimulus probability and simple reaction time. Nature, 215(5103), 895 DOI: 10.1038/215895a0 6049759

[16] Greiter, L., Strauch, C., & Huckauf, A. (2018). Pupil responses signal less inhibition for own relative to other names. In Proceedings of the 2018 acm symposium on eye tracking research & applications (p. 59). DOI: 10.1038/s41598-018-31551-x

[17] Hess, E. H., & Polt, J. M. (1964). Pupil size in relation to mental activity during simple problem-solving. Science, 143(3611), 1190–1192. DOI: 10.1126/science.143.3611.1190 17833905

[18] Hommel, B., Müsseler, J., Aschersleben, G., & Prinz, W. (2001). The theory of event coding (tec): A framework for perception and action planning. Behavioral and Brain Sciences, 24(5), 849–878. DOI: 10.1017/S0140525X01000103 12239891

[19] JASP-Team, et al. (2018). Jasp (version 0.8. 6)[computer software].

[20] Joshi, S., Li, Y., Kalwani, R. M., & Gold, J. I. (2016). Relationships between pupil diameter and neuronal activity in the locus coeruleus, colliculi, and cingulate cortex. Neuron, 89(1), 221–234. DOI: 10.1016/j.neuron.2015.11.028 26711118 PMC4707070

[21] Kiefer, M., Marzinzik, F., Weisbrod, M., Scherg, M., & Spitzer, M. (1998). The time course of brain activations during response inhibition: evidence from event-related potentials in a go/no go task. Neuroreport, 9(4), 765–770. DOI: 10.1097/00001756-199803090-00037 9559953

[22] Klingner, J. (2010). Fixation-aligned pupillary response averaging. In Proceedings of the 2010 symposium on eye-tracking research & applications (pp. 275–282). DOI: 10.1145/1743666.1743732

[23] Kuchinke, L., Võ, M. L.-H., Hofmann, M., & Jacobs, A. M. (2007). Pupillary responses during lexical decisions vary with word frequency but not emotional valence. International Journal of Psychophysiology, 65(2), 132–140. DOI: 10.1016/j.ijpsycho.2007.04.004 17532075

[24] Mathôt, S. (2018). Pupillometry: Psychology, physiology, and function. Journal of Cognition, 1(1). DOI: 10.5334/joc.18 PMC6634360 31517190

[25] Murphy, P. R., O’Connell, R. G., O’Sullivan, M., Robertson, I. H., & Balsters, J. H. (2014). Pupil diameter covaries with bold activity in human locus coeruleus. Human Brain Mapping, 35(8), 4140–4154. DOI: 10.1002/hbm.22466 24510607 PMC6869043

[26] Murphy, P. R., Vandekerckhove, J., & Nieuwenhuis, S. (2014). Pupil-linked arousal determines variability in perceptual decision making. PLoS Computational Biology, 10(9), e1003854 DOI: 10.1371/journal.pcbi.1003854 25232732 PMC4168983

[27] Naber, M., Stoll, J., Einhäuser, W., & Carter, O. (2013). How to become a mentalist: reading decisions from a competitor’s pupil can be achieved without training but requires instruction. Plos one, 8(8), e73302 DOI: 10.1371/journal.pone.0073302 23991185 PMC3753247

[28] Nieuwenhuis, S., Aston-Jones, G., & Cohen, J. D. (2005). Decision making, the p3, and the locus coeruleus–norepinephrine system. Psychological Bulletin, 131(4), 510 DOI: 10.1037/0033-2909.131.4.510 16060800

[29] Nieuwenhuis, S., De Geus, E. J., & Aston-Jones, G. (2011). The anatomical and functional relationship between the p3 and autonomic components of the orienting response. Psychophysiology, 48(2), 162–175. DOI: 10.1111/j.1469-8986.2010.01057.x 20557480 PMC3797154

[30] Preuschoff, K., ’t Hart, B. M., & Einhäuser, W. (2011). Pupil dilation signals surprise: Evidence for noradrenaline’s role in decision making. Frontiers in Neuroscience, 5, 1–12. DOI: 10.3389/fnins.2011.00115 21994487 PMC3183372

[31] Qiyuan, J., Richer, F., Wagoner, B. L., & Beatty, J. (1985). The pupil and stimulus probability. Psychophysiology, 22(5), 530–534. DOI: 10.1111/j.1469-8986.1985.tb01645.x 4048353

[32] Ratcliff, R. (1993). Methods for dealing with reaction time outliers. Psychological bulletin, 114(3), 510–532. DOI: 10.1037/0033-2909.114.3.510 8272468

[33] Ratcliff, R., Smith, P. L., Brown, S. D., & McKoon, G. (2016). Diffusion decision model: Current issues and history. Trends in Cognitive Sciences, 20(4), 260–281. DOI: 10.1016/j.tics.2016.01.007 26952739 PMC4928591

[34] R-Core-Team, et al. (2013). R: A language and environment for statistical computing.

[35] Richer, F., & Beatty, J. (1985). Pupillary dilations in movement preparation and execution. Psychophysiology, 22(2), 204–207. DOI: 10.1111/j.1469-8986.1985.tb01587.x 3991847

[36] Satterthwaite, T. D., Green, L., Myerson, J., Parker, J., Ramaratnam, M., & Buckner, R. L. (2007). Dissociable but inter-related systems of cognitive control and reward during decision making: evidence from pupillometry and event-related fmri. Neuroimage, 37(3), 1017–1031. DOI: 10.1016/j.neuroimage.2007.04.066 17632014

[37] Schneider, M., Leuchs, L., Czisch, M., Sämann, P. G., & Spoormaker, V. I. (2018). Disentangling reward anticipation with simultaneous pupillometry/fmri. NeuroImage, 178, 11–22. DOI: 10.1016/j.neuroimage.2018.04.078 29733957

[38] Simpson, H. (1969). Effects of a task-relevant response on pupil size. Psychophysiology, 6(2), 115–121. DOI: 10.1111/j.1469-8986.1969.tb02890.x 5345494

[39] Simpson, H., & Hale, S. M. (1969). Pupillary changes during a decision-making task. Perceptual and Motor Skills, 29(2), 495–498. DOI: 10.2466/pms.1969.29.2.495 5361713

[40] Strauch, C., Ehlers, J., & Huckauf, A. (2017). Pupil-assisted target selection (pats). In Ifip conference on human-computer interaction (pp. 297–312). DOI: 10.1007/978-3-319-67687-6_20

[41] Strauch, C., Greiter, L., & Huckauf, A. (2018). Pupil dilation but not microsaccade rate robustly reveals decision formation. Scientific Reports, 8(1), 13165 DOI: 10.1038/s41598-018-31551-x 30177773 PMC6120888

[42] van Olst, E., Heemstra, M., & Ten Kortenaar, T. (1979). Stimulus significance and the orienting reaction In H. Kimmel, E. Van Olst & J. Orlebeke (Eds.), The orienting reflex in humans (pp. 521—547). New York, NY: Lawrence Erlbaum Associates.

[43] Varazzani, C., San-Galli, A., Gilardeau, S., & Bouret, S. (2015). Noradrenaline and dopamine neurons in the reward/effort trade-off: a direct electrophysiological comparison in behaving monkeys. Journal of Neuroscience, 35(20), 7866–7877. DOI: 10.3389/fnbeh.2015.00310 25995472 PMC6795183

[44] Verleger, R., Cäsar, S., Stephanie, B., & Smigasiewicz, K. (2017). On why targets evoke p3 components in prediction tasks: Drawing an analogy between prediction and matching tasks. Frontiers in Human Neuroscience, 11, 497 DOI: 10.3389/fnhum.2017.00497 29066965 PMC5641317

[45] Verleger, R., Grauhan, N., & Śmigasiewicz, K. (2016). Go and no-go p3 with rare and frequent stimuli in oddball tasks: A study comparing key-pressing with counting. International Journal of Psychophysiology, 110, 128–136. DOI: 10.1016/j.ijpsycho.2016.11.009 27845155

[46] Verleger, R., Keppeler, M., Sassenhagen, J., & Śmigasiewicz, K. (2018). The oddball effect on p3 disappears when feature relevance or feature-response mappings are unknown. Experimental Brain Research, 236(10), 2781–2796. DOI: 10.1007/s00221-018-5334-z 30030588

[47] Verleger, R., & Śmigasiewicz, K. (2016). Do rare stimuli evoke large p3s by being unexpected? a comparison of oddball effects between standard-oddball and prediction-oddball tasks. Advances in Cognitive Psychology, 12(2), 88 DOI: 10.5709/acp-0189-9 27512527 PMC4975594

[48] Wolfe, J. M., Palmer, E. M., & Horowitz, T. S. (2010). Reaction time distributions constrain models of visual search. Vision Research, 50(14), 1304–1311. DOI: 10.1016/j.visres.2009.11.002 19895828 PMC2891283

[49] Zekveld, A. A., Koelewijn, T., & Kramer, S. E. (2018). The pupil dilation response to auditory stimuli: Current state of knowledge. Trends in hearing, 22, 1–25. DOI: 10.1177/2331216518777174 PMC6156203 30249172

